# Dendritic cell vaccines: Current research progress, challenges, and opportunities

**DOI:** 10.1016/j.gendis.2025.101913

**Published:** 2025-10-31

**Authors:** Shilin Zhang, Shanzhao Mo, Wenxing Huang, Dani Zhong, Xiaomei Yang, Shenxia Xie, Aiqun Liu, Fengzhen Mo, Xianing Huang, Heng Liu, Yangzi Li, Xiaoling Lu

**Affiliations:** a College & Hospital of Stomatology, Guangxi Medical University, Nanning, Guangxi 530021, China; b The Second Affiliated Hospital of Guangxi Medical University, Nanning, Guangxi 530021, China; c Guangxi Key Laboratory of Nanobody Research, Nanning, Guangxi 530021, China; d Department of Gastroenterology and Respiratory Medicine & Endoscopy Center, Guangxi Medical University Cancer Hospital, Nanning, Guangxi 530021, China; e School of Basic Medical Sciences, Guangxi Medical University, Nanning, Guangxi 530021, China; f Pharmaceutical College, Guangxi Medical University, Nanning, Guangxi 530021, China; g Department of Oncology, The Affiliated Suzhou Hospital of Nanjing Medical University, Suzhou, Jiangsu 215001, China

**Keywords:** Cancer vaccine, Combination therapy, Dendriticcell vaccines, Immunosuppression, Immunotherapy

## Abstract

This review examines the advancements in cancer immunotherapies, particularly focusing on dendritic cell (DC)-based vaccines developed through *in vitro* methods. DCs are essential for connecting innate and adaptive immunity and serve as powerful antigen-presenting cells. They play an essential role in the anti-tumor immune response by activating cytotoxic T lymphocytes and natural killer cells. DC vaccines, which involve engineering DCs to express tumor-associated antigens and administering them to patients, potentially enhance the T-cell-mediated destruction of tumor cells. The review details the progression of DC vaccine preparation from simple antigenic peptide pulsing to advanced genetic modification and cell fusion techniques. It discusses the use of envelope fusogenic membrane glycoproteins and chemical agents, such as polyethylene glycol, to facilitate the fusion of DCs with tumor cells, creating fusion cell vaccines that exhibit anti-tumor efficacy in both preclinical and clinical settings. Recent developments of DC vaccines have utilized alternative vectors, addressing some limitations of previous vaccine generations. Additionally, the review examines the integration of DC vaccines with other immunotherapies to combat tumor-induced immunosuppression. Despite their potential, DC vaccines face challenges that necessitate further refinement of therapeutic strategies and clinical validation. In conclusion, this review underscores the pivotal role of DC vaccines in cancer therapy and elucidates ongoing endeavors to augment their efficacy via combination therapies and advanced preparation techniques.

## Introduction

Currently, the focus of anti-tumor research is cancer immunotherapies (CIs). These include dendritic cell (DC) vaccines, immune checkpoint inhibitors (ICIs), and chimeric antigen receptor (CAR) T cell immunotherapy,^1^ with DC vaccines representing a promising form of anti-tumor immunotherapy. DC vaccines employ various methods to induce DCs to express tumor-associated antigens (TAAs) on their surface. This process is completed *in vitro*; the treated DCs are then inoculated into the patient, enhancing the T-cell-mediated destruction of tumor cells.

DCs have been the subject of extensive research since their initial identification by Steinman et al^2^ in 1973. Acting as sentinels of the human immune system, they link innate and adaptive immunity and are considered the most effective antigen-presenting cells (APCs).^3^ The primary types of DCs include plasmacytoid dendritic cells (pDCs), conventional dendritic cells (cDCs), migratory DCs, monocyte-derived dendritic cells (Mo-DCs), and Langerhans cells,^4^^,^^5^ which are prevalent in all human tissues except the brain.

DCs are vital APCs in human-specific immunity, proficient in activating Th cells, cytotoxic T lymphocytes (CTLs), and natural killer cells. Activated Th1 cells secrete interferon-gamma (IFN-γ) and tumor necrosis factor alpha (TNF-α) to kill tumor cells and release interleukin-2 (IL-2) to facilitate CTL activation and proliferation, while enhancing natural killer cell activity, thereby playing a crucial role in anti-tumor immune responses.^6^^,^^7^ Upon recognizing tumor cells, DCs present processed tumor antigens to T cells via the peptide-major histocompatibility complex (pMHC), which serves as the initial activation signal. Co-stimulatory molecules, such as CD80 and CD86, expressed abundantly on the surface of DCs, provide the second signal to activate T cells. Stimulated by these signals, naive T cells become specialized CTLs,^8^ resulting in a targeted anti-tumor effect. However, the functionality of DCs within the tumor microenvironment (TME) can be significantly reduced, especially when tumor cells exhibit low MHC-I expression, hindering DCs' ability to present tumor antigens and activate CTLs effectively.^9^ The development of DC vaccines aims to address these challenges, and since their introduction, DC vaccines have demonstrated some potential.^10^

In this review, we provide a comprehensive overview of existing DC vaccines, including those loaded with antigenic peptides, mRNA-modified DC vaccines, and DC/tumor fusion cell vaccines. We conduct an in-depth analysis of the efficacy and challenges associated with current DC vaccines and assess the potential for combination therapy with DC vaccines. This analysis prompts a focus on integrating DC vaccines with other antitumor therapies.

## Peptide-pulsed DC vaccines

The typical approach to creating DC vaccines includes activating Mo-DCs with tumor antigens to effectively capture and present the tumor's antigenic data, thereby boosting the body's anti-tumor response. Sipuleucel-T, developed by Dendreon in the United States, is the first DC vaccine approved by the US FDA for treating patients with metastatic prostate cancer. This vaccine sensitizes DCs with recombinant prostatic acid phosphate.^11^ Depending on the source of the loaded antigen, vaccines utilizing tumor antigen-sensitized DCs can be classified as whole antigen-sensitized DC vaccines and antigen-peptide-sensitized DC vaccines.

Whole-cell antigens are generally derived from tumor cell lysates, obtained through methods such as chemical agents, freeze-thaw cycling, sonication, hyperthermia inactivation, and ultraviolet irradiation. DC vaccines prepared using tumor cell lysates are loaded with a broad range of antigens, including TAA and tumor-specific antigen (TSA).^12^ This method provides access to a wider antigenic profile and overcomes the limitations of targeting a single antigen. However, it carries the risk of triggering an autoimmune response in patients. In contrast, antigenic peptide-sensitized DC vaccines are prepared using peptide-pulsed DCs that target specific antigens expressed on tumor surfaces. While this method provides a more focused strategy, its effectiveness against tumors is greatly diminished if the tumor antigen undergoes mutation, making preclinical screening crucial.

The specific anti-tumor effect induced by each TAA and TSA on tumor cell surfaces is weak,^13^ leading some researchers to use sequencing and mass spectrometry to identify neoantigens on tumor surfaces for developing a neoantigen-pulsed DC vaccine (Neo-DCVac).^14^ Neoantigens, abnormal proteins or peptides presented as MHC on the cell surface following genetic mutations in the exons of tumor cells, exhibit strong tumor specificity and serve as critical markers for the immune recognition of tumor cells. Neo-DCVac demonstrates strong immunogenicity and specificity, with minimal off-target effects and no impact on normal cells, making it an ideal DC vaccine.^15^ Beyond conventional methods, Neo-DCVac can also be prepared *in vivo*. Tang et al^16^ developed an engineered tumor vaccine called ePAC, which is constructed by integrating genetically encoded neoantigens into endogenous virus-like particles self-assembled from mammalian-derived PEG10 protein, with surface modification of CpG-ODN adjuvant. The vaccine bypasses the *in vitro* induction of Mo-DCs. This vaccine effectively targets and transports neoantigens to DCs *in vivo*, promotes their maturation, generates neoantigen-specific T cells, and, when combined with anti-T-cell immunoglobulin and mucin domain-containing protein 3 (TIM-3) therapy, shows significant anti-tumor efficacy.

To enhance the clinical efficacy of peptide-pulsed DC vaccines, researchers have proposed several strategies. For instance, injecting a small dose of recombinant human granulocyte colony-stimulating factor (rhG-CSF) into patients has been shown to increase the number of antigen-specific CTLs produced by DC vaccine injection, as G-CSF mobilizes myeloid precursors from bone marrow into peripheral blood and promotes their differentiation into functionally mature DCs, while up-regulating DC co-stimulatory molecule expression and antigen-presenting capacity.^17^ Dasyam et al^18^ designed DC-based vaccines loaded with long peptides from NY-ESO-1 and natural killer cell agonist α-galactosylceramide for treating advanced malignant melanoma in a clinical trial. However, the specific T-cell responses were not significantly enhanced, although natural killer cell activity increased.

Peptide-pulsed DC vaccines have demonstrated considerable therapeutic potential when combined with conventional anti-cancer treatments. Chemotherapy reduces the number of immunosuppressive cells, such as regulatory T cells, by decreasing lymphocytes,^19^ and enhances tumor sensitivity to CTL killing by altering tumor cell phenotypes and genetics.^20^ The combination of DCVAC/LuCa and chemotherapeutic agents in treating non-small cell lung cancer is well tolerated and extends survival, with prior administration of chemotherapy proving more beneficial for the DC vaccine's effectiveness.^21^ When the DC vaccine loaded with melanoma tumor markers was used in conjunction with the chemotherapeutic agents dacarbazine and fotemustine for treating patients with advanced malignant melanoma, patient survival extended somewhat, and the DC vaccine exhibited a good safety profile.^22^ The DC vaccine in combination with chemotherapeutic agents has been employed in treating ovarian cancer,^23^ metastatic endometrial cancer,^24^ and prostate cancer.^25^ This combination has yielded positive clinical outcomes in these cancers, suggesting that combining the DC vaccine with agents that can mitigate immunosuppression will likely guide future developments in this therapy.

## Engineered DCs

**Figure 1:**
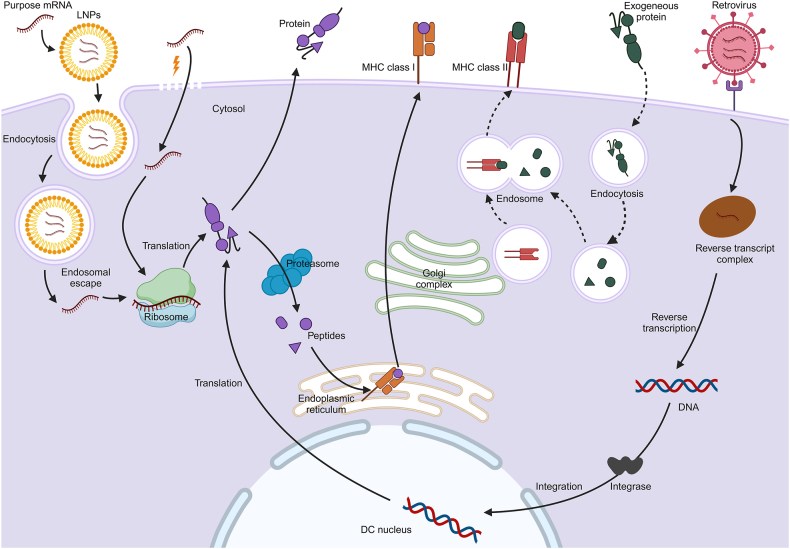
Pharmacological mechanism of adaptive immune responses induced by mRNA-based dendritic cell (DC) vaccines. mRNA is transfected into DCs using lipid nanoparticles (LNPs) or electrofection, translated by ribosomes to either be released from the cell or degraded by proteasomes for loading onto MHC class I molecules, and presented to CD8^+^ T cells (solid line arrow, left). mRNA is reverse-transcribed and integrated into the cell nucleus following DC infection by a retrovirus (solid line arrow, right). MHC class II pathway works for the presentation of exogenous proteins (dashed line arrow).

In addition to stimulating Mo-DCs with antigens to induce maturation and antigen carriage, Mo-DCs can be genetically altered by inserting gene fragments that encode TAAs, TSAs, cytokines, or chemokines. This modification allows Mo-DCs to stably express TAAs on their surface, thereby efficiently inducing a tumor-killing immune response (Fig. 1).

Vaccine preparation involves the use of virus-infected DCs, where genes encoding TAAs are inserted into viral vectors,^13^ such as recombinant, replication-deficient, or attenuated viruses (*e.g.*, lentiviruses, adenoviruses, *etc*.). These DCs then express TAAs following infection. The correlation between the rate of viral infection and TAA expression on DCs' surfaces has been established. Kim et al^26^ designed an adenoviral vector targeted at DCs that significantly enhanced both the infection efficiency of DCs and the expression rate of TAA on their surfaces. However, the general specificity of the virus is poor, and the infection rate is low, limiting both development and clinical application. More recently, the *in vitro* assembly of monodisperse virus-like particles using purified podoplanin proteins from plant viruses has been explored. This approach not only increased maturation markers such as CD80, CD86, and MHC-II on cultured immature DCs but also enhanced RNA replication and significantly raised the number of antigen-specific T-cells in mice.^27^

Additionally, the liposome transfection method enables mRNA transfection into DCs. Markov et al^28^ employed cationic liposomes, composed of novel cationic lipids and lipid adjuvants, to transfect plasmid DNA and mRNA into DCs, producing DC vaccines *in vitro*. Animal experiments showed a transfection efficiency of 57% using this novel cationic liposome. The efficient introduction of mRNAs encoding tumor antigens into DCs remains under investigation. Cooler et al^29^ developed a new mRNA delivery system using polylactic acid nanoparticles and cationic cell-penetrating peptides as coagulants. In this system, enhanced mRNA uptake by DCs was facilitated through clathrin-mediated endocytosis, resulting in high mRNA expression within DCs. Furthermore, this coagulant was found to induce DC activation and maturation and increase their antigen processing capability, thus providing a robust platform for DC vaccine development.

Besides these methods, genes encoding TAAs, cytokines, or chemokines can also be introduced by electrotransfection, with DCs overexpressing these genes forming a DC vaccine that either presents antigenic peptides to activate CTLs or continuously secretes relevant cytokines to bolster the immune response. TriMixDC-MEL, a DC vaccine created by electro-transfecting a fusion mRNA encoding a full-length melanoma-associated antigen (MAA) along with a human leukocyte antigen (HLA) class II molecule mRNA into a DC, presents the complete peptide of MAA and circumvents the HLA restriction of prior peptide vaccines. Phase I clinical trial results indicated that 15 advanced melanoma patients could receive TriMixDC-MEL, resulting in high tolerance, two cases of complete remission, and two cases of partial remission.^30^ The phase II clinical trial revealed a one-year survival rate of only 35 % in the control group, compared with 71% in the TriMixDC-MEL group, nearly doubling the survival rate.^31^ Despite numerous proposals to enhance the transfection method, the mRNA transfection rate remains unsatisfactory, impacting DC activity. The lipid nanoparticle-based COVID-19 vaccines developed by BioNTech/Pfizer and Moderna have demonstrated effective mRNA delivery,^32^ and lipid nanoparticles offer advantages, such as protection of mRNA from degradation, co-delivery with adjuvants, and straightforward synthesis,^33^ suggesting their potential as a viable method for mRNA delivery in DC vaccines. Moreover, the risk of target genes integrating with DC genomes^34^ and the limited clinical efficacy of mRNA-based DC vaccines pose significant challenges.^35^

## DC/tumor fusion cell vaccines

**Figure 2:**
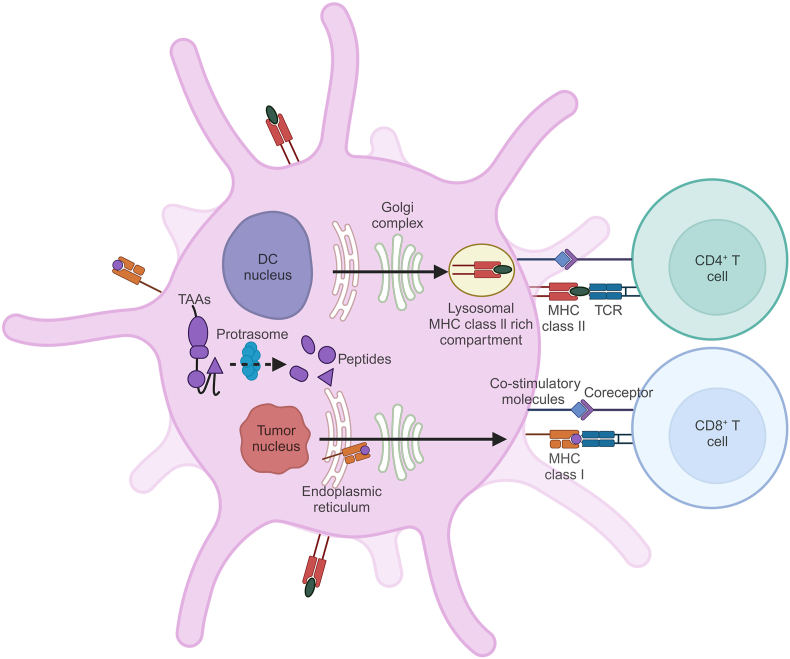
Antigen processing and presentation by dendritic cell (DC)/tumor fusion cells. DC/tumor fusion cells degrade tumor-associated antigens (TAAs) from whole tumor cells via the proteasome (dashed line arrow), and antigenic peptides are loaded onto MHC class I molecules in the endoplasmic reticulum to form peptide-MHC class I complexes, which are then expressed on the surface of fusion cells for presentation to CD8^+^ T cells. The fusion cells also form peptide-MHC class II complexes (solid line arrow) and present them to CD4^+^ T cells. TCR, T cell receptor.

In the 1990s, Gong et al^36^ proposed a novel immunotherapy involving DC/tumor cell fusion for processing and presenting tumor antigens. Mo-DCs with antigen-presenting functions were fused with tumor cells harboring specific antigens to form heterokaryons, *i.e.*, DC/tumor fusion cells, in which cytoplasms were fused while nuclei remained separate. These fusion cells retain intact TAAs from the tumor and MHC class I and II molecules from DCs,^37^ express co-stimulatory molecules and adhesion molecules on their surfaces, and are highly immunogenic and specific,^38^ capable of inducing specific tumor-killing immunity and the production of tumor-specific CTLs^39^ (Fig. 2). Present techniques for creating DC/tumor fusion cell vaccines involve electrofusion, viral fusion, and chemical fusion.

When a cell is subjected to a high-intensity external electric field, the transmembrane potential increases. As the external electric field intensifies, the transmembrane potential gradually exceeds the resting transmembrane voltage in its physiological state, resulting in the formation of pores in the cell membrane, a phenomenon known as electroporation. Cell electrofusion, an application of reversible electroporation, is marked by low fusion efficiency and poor stability.^40^ Rems et al^41^ suggest that reducing pulse duration to nanoseconds, compared with conventional microsecond pulses, lowers the high cellular mortality associated with fusing cells of different sizes. The microelectrode arrays designed by Hu et al^42^ not only enhance electrofusion efficiency but also minimize the occurrence of irreversible electroporation. The adoption of bipolar pulses for electrofusion reduces membrane damage significantly compared with conventional unipolar pulses, tripling the fusion efficiency between myeloma cells and lymphocytes.^43^ Recent studies indicate that hypotonic buffers can enhance electrofusion efficiency by increasing membrane tension and enlarging the cell contact zone, thus improving electrofusion outcomes by adjusting properties, such as osmotic pressure, conductivity, and ionic concentration.^44^^,^^45^ Marko et al^45^ have merged gene electrotransfer with cell electrofusion into a single-step process, broadening the potential for DC/tumor fusion cell vaccine development.

Envelope fusogenic membrane glycoproteins (FMGs) are critical components of viral-initiated cellular infections, mediating fusion between viruses and target cells.^46^ The high fusogenicity of FMGs has facilitated fusion between Mo-DCs and tumor cells.^47^ Phan et al^47^ co-cultured B16 melanoma cells transfected with VSV-G FMGs, successfully creating fusion cells that retained the ability to migrate to lymph nodes, achieving a fusion rate of 38%. In another approach, Kazuya et al^48^ used ultraviolet-inactivated hemagglutinating virus of Japan (HVJ), which retains fusion activity but lacks viral replication capacity, to induce fusion of DC and B16 cells with a fusion efficiency of approximately 25%. Yanai et al^49^ also successfully prepared a fusion cell vaccine that inhibited tumor growth by fusing DC with mouse fibrosarcoma cells, achieving a 20% fusion rate using HVJ-E. However, it has been shown that virus-mediated cell fusion results in the formation of large multinucleated syncytia, which subsequently die via autophagic mechanisms.^50^

Polyethylene glycol (PEG), a chemical membrane destabilizer, has been used since the 1990s to fuse tumor cells with DCs to create fusion cell vaccines and was initially demonstrated in cancer therapy.^36^ With the presence of cations such as Ca^2+^, PEG binds tightly to acidic lipid bilayers, alters the arrangement of membrane molecules at the contact point, dehydrates the contact area, and promotes fusion through positive osmotic pressure. The primary advantage of using PEG for cell fusion is its simplicity and convenience. Due to variations in cell sensitivity to PEG, optimal fusion conditions are attained by adjusting the molecular weight of PEG, contact reaction time, and temperature.^51^ Unlike electrofusion, PEG-induced fusion is a dynamic process; short-term culturing of Mo-DCs and tumor cells can increase fusion efficiency and prevent the overgrowth of unfused tumor cells.^52^ Zhang et al^53^ used 50% PEG 1450 to induce DC/osteoclast fusion cells and incorporated polymeric nanomaterial poly(lactic-co-glycolic) acid to prepare fusion cell membrane nano-vaccine with potent immune activation. Similarly, fusion cells of DCs and human breast cancer cells (MDA-MB-231), designated as DC fusion cells/MDA-MB-231(Gal+), exhibited effective T-cell stimulatory activity and demonstrated strong anti-tumor properties.^54^ Studies have indicated that cell fusion efficiency is closely linked to anti-tumor immunity.^52^ He et al^55^ and Yang et al^56^ added diluted type I collagen to the DCs and tumor cell fusion system, stabilizing the fusion membrane and significantly enhancing fusion efficiency. Compared with electrofusion and viral fusion, the PEG fusion method avoids complexity and cytotoxicity, thus holding a significant advantage in the preparation of cell-fusion vaccines.

DC fusion cell vaccines have demonstrated strong anti-tumor immunity in both cellular and animal experiments and have yielded significant results in clinical studies. Clinical trials of these vaccines initially targeted melanoma patients and have since extended to patients with solid tumors and hematological malignancies.^57^ For instance, multiple myeloma patients vaccinated with increasing doses of autologous DCs/myeloma fusion cell vaccines experienced stable and tolerable responses, with adverse reactions not exceeding grade 2, typically transient reactions at the injection site.^58^ A phase II trial involving patients with stage IV renal cell carcinoma indicated that eight patients exhibited tumor regression or maintained stable disease for at least 16 weeks, achieving a 1-year progression-free survival rate of 25%.^59^ These findings underscore the feasibility of generating DC/tumor fusion cell vaccines. A recent phase II trial in multiple myeloma confirmed tumor-specific immunity in patients who received a DC/myeloma fusion cell vaccine.^39^ In addition, DC fusion cell vaccines for osteosarcoma, ovarian cancer, and hepatocellular carcinoma have shown anti-tumor activity in animal models and preliminary clinical trials. However, the clinical trials for DC fusion cell vaccines have not yet reached the effectiveness of other immunotherapies, necessitating further clinical development.

## pDC-derived vaccines

The functionality of Mo-DCs is compromised by several factors, such as pre-vaccine preparation induction culture, leading to functional exhaustion and diminished antigen-presenting and T-cell activation abilities after prolonged *in vitro* culture.^60^ Recent studies indicate that Mo-DCs, whether cryopreserved or not,^61^ exhibit reduced antigen uptake and T-cell activation capabilities compared with primary DCs, resulting in the ineffectiveness of Mo-DC-based vaccines.^62^ Preliminary clinical trials have demonstrated that next-generation DC vaccines derived from natural DC subpopulations are safe, feasible, and potentially effective.^63^ Certain natural DC subpopulations, which do not require *in vitro* maturation, offer superior functionality and are considered preferable alternatives to Mo-DCs.^64^^,^^65^ Current techniques facilitate the rapid isolation of natural DCs,^63^ meeting production standards for cost-effective, large-scale supply.^66^ Notably, specific DC subpopulations excel in antigen presentation and initiating CTL responses to MHC molecules,^67^ including pDCs, which are particularly effective in type I IFN responses.^68^

In addition to secreting type I IFN, pDCs produce cytokines, such as IL-6, IL-12, C-X-C motif chemokine ligand 8 (CXCL8), CXCL10, C–C motif chemokine ligand 3 (CCL3), and CCL4, contributing to anti-tumor immunity. Enhanced natural killer cell activation via IFN-I, IL-12, and IL-18 and increased expression of sFasL, perforin, and IFN-γ induce apoptosis in target cells bearing death receptors (TRAIL-R1, TRAIL-R2, or FAS).^69^ pDCs also directly kill tumor cells through TNF-related apoptosis inducing ligand (TRAIL) and granzyme B-dependent pathways.^70^ High expression of MHC class II molecules along with co-stimulatory molecules CD40, CD80, and CD86 enhances the antigen presentation capabilities of pDCs. By secreting IFN-I and IL-12, pDCs activate and effectuate CD8^+^ T cells and polarize CD4^+^ T cells towards a T helper 1 (Th1) phenotype.^71^ The influence of pDCs on B cell activation, plasminogen, and antibody secretion has also been documented.^72^

Three clinical trials using pDCs as anti-cancer vaccines have been conducted, showing that next-generation DC vaccines derived from a subpopulation of natural DCs are safe, feasible, and clinically effective (Table 1). Tel et al^73^ developed DC vaccines using autologous pDCs loaded with TAA peptides, resulting in specific CD8^+^ T cell responses and detectable IFN responses in some vaccinated melanoma patients. A phase Ib clinical trial of GeniusVac-Mel4s, a vaccine constructed from human pDCs lineage cells,^74^ confirmed these results, demonstrating a favorable safety profile and inducing strong antigen-specific T-cell responses in patients with metastatic melanoma. Furthermore, this pDC vaccine has shown remarkable results in treating not only melanoma but also advanced prostate cancer. In a phase IIa clinical trial for prostate cancer, expression of the target TAA was detected in over 70% of the patients, with specific T-cell responses observed in some, and vaccines derived from both cDC2s and pDCs showed cancer inhibition.^75^

**Table 1:** Summary of current clinical trials with pDCs-derived vaccines.

Cancer Type	Phase	Interventions and combinations	Doses	Patients	ClinicalTrials.gov ID	Clinical Outcomes(Median PFS, OS)	DOI
Stage IV Melanoma	I	autologous pDCs loaded with tumor antigen-associated peptides	three times biweekly	melanoma (tyrosinase/gp100+, ≥20% cells)	NCT01690377	PFS: 4.0 months OS:22.0 months	10.1078-0432.CCR-15-2205; 10.1158/0008-5472.CAN-12-2583
Stage III Melanoma	II	autologous cDC2s and/or pDCs loaded with tumor peptides and overlapping peptide pools	three times biweekly	age 18–75, stage III melanoma (histologically confirmed), with complete resection + RLND ≤12 weeks before study	NCT02574377	PFS: 19.4 months, IFNγ-producing T-cells in 64% (9/14)patients	2162402X.2021.2015113
Chemo-naive Metastatic Castration-resistant Prostate Cancer	IIa	tumor peptide-loaded myeloid and/or plasmacytoid dendritic cells	three times biweekly	HLA-A∗0201+ patients, no prior immunotherapy, taxanes (docetaxel/cabazitaxel), or denosumab	NCT02692976	rPFS: 9.5 months; TAA-specific skin T cells (tetramer^+^/dextramer^+^): 71% (15/21)	10.1186/s40425-019-0787-6
Melanoma	Ib	PDC line from leukemic cells, loaded with multiple melanoma antigens	once weekly for 3 weeks	unresectable metastatic melanoma (stage IIIC/IV), HLA-A∗0201+, ECOG PS < 3, ≥1L systemic therapy failed	NCT01863108	safe and well-tolerated long-term	10.1080/2162402X.2020.1738812
Metastatic Endometrial Cancer	II	myeloid and plasmacytoid DC (nDC) are loaded with tumor lysate and MUC1 and survivin PepTivators	once every 3 weeks	stage IV/metastatic endometrial carcinoma (endometrioid/serous/carcinosarcoma) with Survivin + MUC1+ tumor expression	NCT04212377	PFS:10 months OS: 23 months	10.3389/fimmu.2024.1368103
Non-small-cell Lung Cancer	I/II	PDC∗lung01: seven lung tumor antigen peptide preparations	once every 3 weeks	HLA-A∗02:01 positivity	NCT03970746		
stage IIIB/C melanoma	III	autologous nDCs loaded with tumor peptides and overlapping peptide pools or placebo	3 biweekly intranodal injections, repeated after 6 and 12 months	patients with resected stage IIIB/C melanoma	NCT02993315	PFS: 12.7 months, 2-year OS rate 84.7%	10.1038/s41467-024-45358-0

A current uncertainty in these clinical studies is the advantage of specific DC subpopulations over others; thus, comparing the efficacy of DC vaccines based on different DC subpopulations in cancer patients will be essential in the near future. The most effective DC vaccines may include multiple DC subpopulations to utilize their complementary functions and facilitate crosstalk between innate and adaptive immune cells,^76^ suggesting that combining multiple DC subpopulations could be a viable strategy to enhance the efficacy of DC vaccines.

## DC-derived exosome vaccines

It is recognized that DCs efficiently present antigens to maximize T cell activation. In DC vaccines, DCs serve more effectively as carriers than as APCs. Conversely, exosomes are viable vectors, and DC-derived exosomes (Dex) notably excel in inducing CTLs and combating immunosuppression.^77^ DC endosomes can merge with the plasma membrane to release membranous vesicles, termed Dex, ranging from 30 to 150 nm in diameter, which encompass protein metabolites and nucleic acids.^78^ Dex, carrying pMHCI, pMHCII, and co-stimulatory molecules, can present antigens and activate CD8^+^ T cells,^79^ and are involved in various biological processes, including immune response, antigen presentation, cellular differentiation, and tumor invasion.^80^ As inert vesicles, Dex is more resistant to tumor-mediated immunosuppression, making them a superior cell-free DC vaccine compared with Mo–DC vaccines produced *in vitro*.^81^

Dex exerts its anti-tumor effects through multiple immunomodulatory pathways. Evidence suggests that its surface pMHC transfers antigens to bystander APCs.^82^ APCs phagocytose or internalize a portion of Dex, processing the pMHC for presentation to CD4^+^ and CD8^+^ T cells, triggering specific CTL responses. Co-stimulatory molecules, such as CD86, enhance T-cell activation. Dex also directly fuses with APC cell membranes, transferring its pMHC to the APC surface via heterodimerization,^83^ enabling rapid T-cell activation and tumor antigen targeting without further antigen processing.^84^ In addition, Dex can transfer pMHC to tumor cell surfaces, enhancing tumor cell immunogenicity and intercellular adhesion molecule 1 (ICAM1) expression, which further activates T cells to increase IFN-γ secretion, enhancing tumor cell killing.^85^

Li et al^86^ developed a nano-vaccine platform containing Dex and patient-specific neoantigens for individualized immunotherapy. This approach induced potent CTLs and B cell-mediated immune responses, significantly reducing tumor growth and eliminating lung metastases in therapeutic, prophylactic, and metastatic B16F10 melanoma models, as well as in the MC-38 therapeutic model. Three clinical reports (two phase I and one phase II) of Dex derived from Mo-DCs have been published^87^, ^88^, ^89^ (Table 2). The results indicate that these Dex vaccines are safe and well-tolerated and enhance natural killer cell activity.

**Table 2:** **Summary of current clinical trials with DC-derived exosomes (Dex).** Copyright 2021, MDPI.

Cancer Type	Phase	Exosomes/Antigen	Doses	Patients	Toxicity	Clinical Outcomes	DOI
Advanced Non-small cell lung cancer	I	Exosomes were isolated from autologous MoDCs generated *in vitro*, and loaded with MAGE peptides	once weekly for 4 weeks	13 (9 completed) HLA-A2^+^ stage IIIb and IV NSCLC patients with tumor expression of MAGE3 or MAGE4	Grade 1–2 toxicity	DTH reactivity against MAGE peptides in 3/9; MAGE-specific T cell responses in 1/3 patients examined; increased NK lytic activity in 2/4	10.1186/1479-5876-3-9
MAGE3-expressing advanced melanoma	I	Autologous MoDC-derived exosomes were loaded with MAGE3 peptides	once weekly for 4 weeks	15 stage IIIb and IV, HLA-A1^+^, B35^+^ or HLA-DPO4^+^ patients	Only grade 1 toxicity	No detectable MAGE3-specific CD4 and CD8 T cell responses; restored NKG2D expression and NKG2D-dependent function of NK cells in 7/14 patients; 1/15 partial responses	10.1371/journal.pone.0004942 and 10.1186/1479-5876-3-10
Advanced colorectal cancer	I	Exosomes from patient ascites ± GM-CSF, ASexos contained CEA with no additional antigen loading.	once weekly for 4 weeks	40 HLA-A2^+^CEA^+^ stage III and IV CRC patients	Grade 1–2 toxicity	DTH induction in both groups, and CEA-specific CTL responses were observed in ASexo + GM-CSF group. 1 stable disease and 1 minor response in ASexo + GM-CSF group	10.1038/mt.2008.1
Non-small cell lung cancer	II	IFN-γ-matured autologous MoDCs were loaded with both MHCI and MHCII tumor epitopes.	exosome immunization in 1, 2 and 3 week intervals in a maintenance immunotherapy regime	26 (22 HLA-A2^+^ stage IIIb and IV NSCLC patients	1/22 grade 3 hepato-toxicity	No detectable induction of antigen-specific T cell responses; increased NKp30-dependent NK cell function; 7 patients (32%) with progression-free survival at 4 months after chemotherapy cessation; no objective tumor response according to RECIST criteria	10.1080/2162402X.2015.1071008

**Figure 3:**
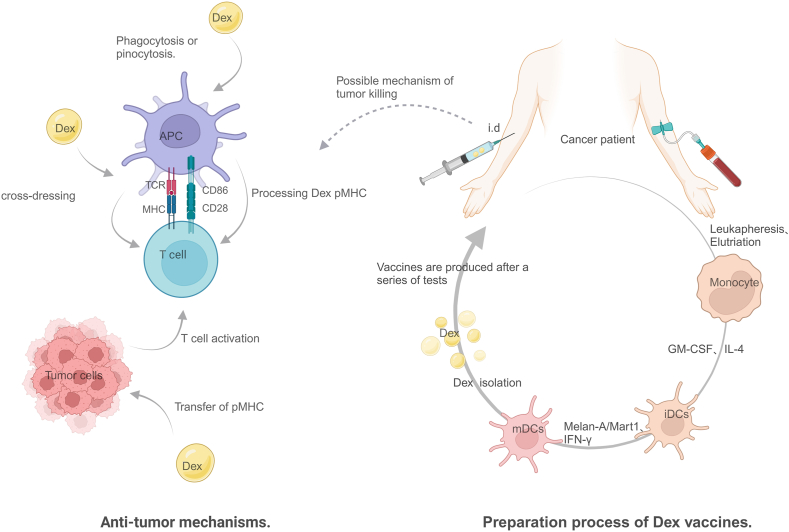
Preparation process and anti-tumor mechanism of Dex vaccines.

Researchers used exosomes derived from immature dendritic cells (imDex) in phase I anti-tumor clinical trials. They constructed an imDex-based DC vaccine by directly loading TAAs into exosomes. Preliminary results showed its ability to activate natural killer cells, but not T cells.^87^ To overcome this limitation, Nathalie et al developed a second-generation exosome for phase II trials by interferon-γ treatment^89^ (Fig. 3). Evidence suggests that the maturation stage of DCs affects the phenotype of their secreted Dex. Exosomes derived from mature dendritic cells (mDex) express higher levels of co-stimulatory molecules, such as CD40, CD80, CD86, and ICAM.^90^ The second-generation Dex vaccine was prepared by isolating immature DCs from cancer patient collections, loading Melan-A/Mart1 peptides and IFN-γ into MHC class I and II antigenic peptides, inducing immature DCs to mature, and isolating exosomes from the culture medium through ultracentrifugation. Immunological characterization and quality control of the exosomes revealed that they were loaded with Melan-A/Mart1 and could activate CTLs.^91^

However, antigen-specific T-cell responses were not detected in all Dex vaccine clinical trials. This may be because the antigen on Dex is insufficient to initiate specific T-cell activation *in vivo*. However, a study found that protein-loaded Dex could trigger allogeneic CD8^+^ T-cell responses *in vivo*,^92^ suggesting the potential of these allogeneic Dex as non-personalized cancer vaccines that are not restricted by MHC. Nevertheless, protein-loaded Dex vaccines have not yet been tested in clinical settings.^93^

## DC vaccines in combination with other immunotherapies

More than two hundred clinical trials of DC vaccines have been completed.^94^ Although their safety is well-established, their clinical efficacy remains limited.^95^ Several studies highlight tumor-mediated immunosuppression as the primary barrier to success.^96^ DC function is often impaired by TME.^97^ Lactate binding to GPR81 in the TME down-regulates MHC-II expression on dermal DCs, thereby impairing their antigen-presenting capacity to T cells.^98^ Studies demonstrate that lactate derived from tumors activates the sterol regulatory element-binding protein 2 (SREBP2)-mevalonate metabolic axis, facilitating the phenotypic conversion of conventional DCs into immunosuppressive mature regulatory DCs.^99^ IDO (indoleamine 2,3-dioxygenase)-expressing DCs constitute a key, crucial immunosuppressive mechanism.^100^ IDO-mediated tryptophan catabolism reduces tryptophan levels and increases kynurenine, consequently inhibiting T cell proliferation. Simultaneously, IDO activation suppresses T cell responses and imparts tolerogenic functional characteristics to DCs.^101^^,^^102^ The activation of the β-catenin signaling pathway in tumor cells down-regulates CCL4 chemokine and suppresses CXCL9/10 secretion, significantly impairing cDC1 tumor infiltration capacity, thereby blocking effective CD8^+^ T cell recruitment into the TME.^103^ It can also cause abnormal differentiation of DC precursors, reducing DC numbers and inhibiting antigen presentation by mature DCs.^104^ Additionally, DC phenotypic changes can allow tumor cells to escape immune detection.^105^^,^^106^ Other immune cells, such as regulatory T cells, also interact with DCs to promote immune tolerance.^107^ Tumor cells can impair DC cross-activation, depleting CD8^+^ T cells and significantly reducing CTL production.

Although clinical trials have shown limited success, DC vaccines are vital for cancer patients as they provide a powerful way to produce targeted anti-tumor immunity.^108^ Recent studies have demonstrated that cDC1s activate CD8^+^ T cells through cross-presentation of tumor antigens and directly stimulate CD4^+^ T cells via MHC-II. Additionally, they orchestrate the interaction among these immune cells via CD40-CD40L signaling-mediated “licensing” effects. Furthermore, cDC1s produce IL-12, which subsequently activates CD4^+^ T cells and natural killer cells, thereby enhancing the anti-tumor efficacy of other immunotherapeutic approaches.^109^^,^^110^ There is a strong need to develop more refined therapeutic regimens that combine DC vaccines with other treatments, particularly those that counteract immunosuppression, to improve clinical outcomes.^111^^,^^112^ Tumor recurrence remains a major challenge in cancer therapy. When patients are prone to recurrence after a single treatment, combining DC vaccines with other immunotherapies may offer a more effective solution.

## Combinations with ICIs

Inhibitory immune checkpoint ligands, often overexpressed in solid tumors, induce immune suppression in the TME. For example, programmed cell death ligand 1 (PD-L1), the ligand for the programmed cell death-1 (PD-1) receptor, inhibits T-cell activation by binding to PD-1. ICIs are monoclonal antibodies that block these immune checkpoints, preventing tumor cells from depleting CD8^+^ T cells^113^^,^^114^ and enhancing anti-tumor immunity. ICIs have been developed over many years and are now a well-established immunotherapy. Several tumor-related immune checkpoint molecules, including cytotoxic T-lymphocyte antigen 4 (CTLA-4), PD-1/PD-L1, lymphocyte-activation gene 3 (LAG-3), and TIM-3, have been identified as key therapeutic targets for cancer treatment.^115^

ICIs can reduce immunosuppression in the TME and boost the efficacy of DC vaccines,^116^^,^^117^ resulting in better clinical results.^118^^,^^119^ Ding et al^120^ designed a clinical trial combining Neo-DCVac with nivolumab to treat patients with advanced lung cancer. The combination caused no adverse reactions and demonstrated better efficacy than either treatment alone, with Neo-DCVac inducing a renewed immune response in patients receiving nivolumab.^121^ Combining DC vaccines with PD-1 blockers can also promote eosinophilic infiltration, which helps treat ovarian cancer through direct or indirect mechanisms.^121^ Co-injection of chemokines with peptide-pulsed DC vaccines can enhance the anti-tumor effect.^122^ In addition to direct chemokine injection, engineered DC vaccines, such as those using CXCL9 and CXCL10, can be combined with ICIs targeting PD-1 to treat non-small cell lung cancer in mouse models, overcoming drug resistance and establishing systemic tumor-specific immunity.^123^ While most current ICI combinations with DC vaccines target PD-1, Sprooten et al^124^ blocked immunosuppression from PD-L1^+^ tumor-associated macrophages using a PD-L1 blocker, and found that DC vaccines promoted immune responses even against PD-L1^+^ tumor-associated macrophages, significantly inhibiting lung cancer growth in mice.

Despite their therapeutic efficacy, monoclonal antibodies face limitations, such as poor tumor penetration and high cost due to their complex production process.^125^ In recent years, nanobodies have gradually entered the field of tumor immunotherapy due to their natural advantages that compensate for the shortcomings of murine-derived monoclonal antibodies. First discovered in Camelidae, nanobodies contain only one heavy chain variable region in their binding domain,^126^ offering benefits such as low molecular weight, high stability, enhanced antigen recognition, and the ease of constructing multifunctional structures.^127^ Since nanobodies lack Fc fragments and are highly homologous to the human heavy chain variable region, they can effectively avoid complement activation and host-anti-graft reactions often seen with monoclonal antibodies.^128^ Our group developed a PD-1-blocking nanobody (PD-1 Nb20) that blocks PD-1/PD-L1 interactions to reduce CD8^+^ T-cell depletion in the TME. Combining this nanobody with DC/tumor fusion cell vaccines shows great promise in treating cancers, such as lung cancer.^129^

Although ICIs are effective in relieving immunosuppression, the cancers most responsive to DC vaccines, such as glioblastoma and renal cancer, are not sensitive to ICIs targeting PD-1 and CTLA-4.^130^ Further research is needed to identify other cancer types that could benefit from combining ICIs and DC vaccines. Current ICI development focuses on multi-targeted inhibition, and the combination of bispecific or trispecific ICIs with DC vaccines may represent a new direction for future clinical applications.

## Combinations with CAR-T cells

CAR-T cells are engineered by combining CARs with T cells through gene editing.^131^^,^^132^ These CARs bind to tumor antigens and activate T cells to secrete cytokines, perforins, and granzymes that play a key role in tumor destruction.^133^ CAR structure-specific tumor recognition helps overcome immune escape mechanisms, such as MHC molecule down-regulation, and counters immune suppression in the TME.^134^ DC vaccines express TAAs that can be recognized by CARs, enhancing CAR-T cell activation and improving the efficacy of DC vaccines in activating bystander T cells.^135^

A peptide-pulsed DC vaccine loaded with epidermal growth factor receptor pathway substrate 8 (Eps8) significantly boosted CAR-T cell proliferation and increased the proportion of central memory T cells after T cell expansion.^136^ Akahori et al^137^ created a DC vaccine loaded with the Wilm tumor gene-1 (WT1) antigen. When CAR-T cells targeting the WT1/HLA complex were co-treated with this DC vaccine, the activation and proliferation of CAR-T cells were enhanced, improving the targeting efficiency of these cells. Neo-DCVac has been shown to promote CAR-T cell localization to tumor cells and improve CAR-T cell expansion and activation when patient-specific tumor neoepitopes are recognized by CARs.^138^

Traditional CAR structures typically use murine monoclonal antibodies for antigen recognition, but their large size, production complexity, and immune rejection can lead to treatment failure and recurrence.^139^ Nanobodies, with small molecular weight and high penetrating power, are the smallest antibodies known to efficiently recognize and bind to antigenic epitopes. It is low in production cost, simple in structure, chemically stable, and has the dual advantages of high affinity and specificity of small molecules.^128^^,^^140^ In recent years, relevant studies have shown that CAR-T cells prepared based on nanoantibody technology (including targeting CD19, CD20, HER2, CD72, CD105, VEGFR2, PSMA, PD-1, TNF-α, TIM-3, CD13, *etc*.) can effectively kill and remove tumor cells and alleviate the tumor load in mice.^141^, ^142^, ^143^, ^144^, ^145^ Sun et al^146^ constructed nanobody-based CAR-T cells targeting epidermal growth factor receptor variant III (EGFRvIII) and combined them with DC/tumor fusion cell vaccine in a mouse model of glioblastoma. Three hormoblastoma mouse models, namely hormoblastoma mice injected subcutaneously with U251-EGFRvIII or U87-EGFRvIII, and a mouse model of carcinoma *in situ* in which mice were injected with U87-EGFRvIII in the brain. Both showed great potential for combination therapy, with a significant increase in the percentage of CAR-T cells after treatment, smaller tumors in the mice, and an extension of their survival.

While several studies demonstrate that combining CAR-T cells with DC vaccines improves the efficacy of traditional DC vaccines and alters T cell ratios in the TME, clinical trial data remain limited. Further research is needed to determine the optimal timing and regimen for this combination therapy. To ensure safety, the use of nanoantibody-based CAR-T cells may offer a more promising solution.

## Combinations with other therapies

DC vaccines have been effectively combined with other anti-tumor modalities, including oncolytic virotherapy and targeted drugs, enhancing immunotherapy efficacy against the immunosuppressive TME. The novel natural oncolytic virus, alphavirus M1, augments the anti-tumor effects of DC vaccines by increasing CD8^+^ T-cell infiltration in the TME, and simultaneously mitigates the dominant immunosuppressive actions of DC vaccines by down-regulating signal regulatory protein alpha (SIRPα) in DCs and CD47 in tumor cells.^147^ Furthermore, as alphavirus M1 up-regulates PD-L1 in DCs, a triple combination of an anti-PD-L1 antibody with DC vaccine and alphavirus M1 therapy can significantly enhance anti-tumor activity, presenting a promising therapeutic strategy for malignant tumors. Experimental studies have shown that transcription factor T-bet genetically modified DCs promote TME vascular normalization, diminish myeloid-derived suppressor cell infiltration, and significantly decrease regulatory T cell levels, thereby creating a conducive Th1-polarized anti-tumor microenvironment.^148^ Additionally, glucocorticoid-induced tumour necrosis factor receptor-related receptor ligand (GITRL) presented on the surface of DCs augments CTL activation and improves effector T cell resistance to regulatory T cell-mediated immunosuppression.^97^ Commonly used targeted drugs, such as sunitinib, deplete myeloid-derived suppressor cells and mitigate immunosuppression, and, in combination with DC vaccines, induce specific T-cell responses and inhibit tumor growth in treated mice.^149^ However, the number of pertinent clinical trials remains limited, and the efficacy of these combinations has yet to be fully demonstrated.

## Summary and outlook

Since their inception, DC vaccines have become a pivotal tool in immunotherapy, exhibiting substantial progress in research and now boasting a comprehensive theoretical framework and mature preparation processes. The approach to antigen loading in DC vaccines has developed from simple peptide pulsing to genetic modification and cell fusion, expanding the array of markers that can be presented. The carriers of these vaccines have expanded from Mo-DCs to other autologous DC types and DC-Dex, addressing the issue of reduced antigen-presenting capacity in Mo-DCs due to prolonged *in vitro* culturing.

While DC vaccines hold immense potential and are considered a future cornerstone of anti-tumor therapy, clinical trial results have been modestly satisfactory. The principal challenge is tumor-induced immunosuppression in the TME, which includes but is not limited to reducing DC populations, causing T-cell depletion, and up-regulating immunosuppressive cells. Despite these challenges, the ongoing refinement of mechanistic studies has significantly advanced DC vaccines, particularly when combined with other therapies. ICIs relieve TME immunosuppression and T-cell depletion, CAR structures in CAR-T cells recognize DC vaccine antigens to activate bystander T cells, and oncolytic virotherapy along with targeted drugs deplete myeloid-derived suppressor cells, enhancing the immunosuppressive milieu.

The GMP-standardized production of DC vaccines poses multiple challenges concerning scalability, cost-efficiency, and regulatory adherence. The production of personalized DC vaccines under GMP conditions is inherently intricate and costly, especially for autologous therapies like Sipuleucel-T, which face considerable batch-to-batch variability, extended preparation durations, and elevated treatment expenses, significantly limiting their clinical utility.^150^ Despite the potential for scalable production of allogeneic DC vaccines, their clinical application is hindered by challenges, including long-term efficacy durability, HLA restriction, safety issues related to allogeneic antigens, and interference from the patient's immune microenvironment.^151^ Meanwhile, the exploration of biomarkers that can forecast vaccine responses has emerged as an essential approach for improving therapeutic efficacy. The functional status of vaccines can be effectively evaluated by monitoring DC maturation markers (such as MHC molecules and CD80/CD86) and T cell receptor clonality and assessing IFN-γ secretion levels and T-cell phenotypic changes.^152^

Contemporary research primarily concentrates on conventional DC vaccines, highlighting the imperative to expand investigations toward developing innovative DC vaccines capable of activating CD8^+^ T cells *in vivo* and inducing potent anti-tumor immunity. Further investigation into the protocols for integrating DC vaccines with other therapeutic modalities, as well as determining the optimal timing for initiating immunotherapy, could enhance efficacy while minimizing toxicity. Due to their small size, stable properties, and low production costs, nanoantibodies present a promising avenue for future research in combination therapy. Overall, DC vaccines exhibit significant potential in oncology, necessitating ongoing research to optimize these therapies for effective patient treatment.

## CRediT authorship contribution statement

**Shanzhao Mo:** Writing – review & editing. **Wenxing Huang:** Writing – review & editing.

## Funding

The work reported in this article was partly funded by the International (Regional) Cooperation and Exchange Program of the National Natural Science Foundation of China (No. 82220108003), the Guangxi Science and Technology Major Program (China) (No. GuiKe-Research AA24263007), the Guangxi Key and Development Program (China) (No. GuiKe-Research AB21196024), and the Guangxi Key Laboratory of Nanobody Research (China) (No. 21-220-16).

## Conflict of interests

The authors have declared that no competing interest exists.
